# Assesment of *Hypoderma* infestation in a wild population of *Cervus elaphus* from mountains Atlantic ecosystems in southwestern Europe (Spain)

**DOI:** 10.1007/s11259-023-10240-9

**Published:** 2023-10-31

**Authors:** Sara González, Rosario Panadero, María Luisa Del Rio, María Natividad Díez, María del Rosario Hidalgo, Angélica Martínez

**Affiliations:** 1 Department of Animal Health, Parasitology and Parasitic Diseases, Faculty of Veterinary Science, University of León, 24071 León, Spain; 2 Department of Animal Pathology, Animal Health, Faculty of Veterinary Science, University of Santiago de Compostela, 27002 Lugo, Spain; 3 Department of Animal Health, Section of Immunobiology, Faculty of Veterinary Science, University of León, 24071 León, Spain; 4 Department of Biotechnology and Food Science, Faculty of Sciences, University of Burgos, 09001 Burgos, Spain

**Keywords:** *Hypoderma actaeon*, *Cervus elaphus*, Epidemiology, Serology, Spain

## Abstract

Hypodermosis in *Cervus elaphus* was studied in the Riaño Regional Hunting Reserve, Province of León, north-western Spain. One hundred and ten red deer were examined for the presence of warble fly larvae. They were analyzed by PCR analysis of the COI region of mt-DNA and identified as *Hypoderma actaeon*. The prevalence of larvae was 42.7% with a mean intensity of 12.5 ± 18 (range 1–80) warbles/deer infested. The distribution of larvae in the infested animals showed an aggregated/overdispersed pattern (aggregation index = 25.84), where the larvae are not randomly or uniformly distributed, but strongly aggregated among their hosts. Larvae were found in all three states. First and second-instars were observed mainly in the autumn until the end of winter (November-March) and third-instars in late winter until mid-spring (March–May). The adult animals and the males had a higher prevalence than the young and the females, finding statistically significant differences only according to the sex of the animals. Seasonal variations were observed in the prevalence with the highest number of infested animals in winter and autumn, but not in terms of the mean intensity of parasites. Additionally, we assessed the presence of anti-*Hypoderma* antibodies in serum by means of indirect ELISA tests, using a crude larval extract (CLE) and a purified fraction the hypodermin C (HyC) obtained from first instars of Spanish isolates of *Hypoderma lineatum* (cattle). These findings confirm that *H. actaeon* is widely distributed in northern Spain, and provide new information about its chronobiology in mountainous Atlantic ecosystems from southwestern Europe.

## Introduction

Hypodermosis is a subcutaneous myiasis caused by fly larvae (Diptera, Oestridae) belonging to the genus *Hypoderma* with species whose larvae, in general, penetrate and migrate through the host's organs and tissues until they reach the subcutaneous dorsolumbar tissue, originating a nodule or “warble” where they develop at second and third-instar. These, when they are mature, leave the host, pupate in the ground and the adult flies emerge. These parasites have an annual life cycle, affect domestic and wild animals of the Bovidae and Cervidae families and are distributed in the Holarctic ecozone (Zumpt 1965).

The larvae are generally host-specific; only closely related hosts are normally infested by the same species of *Hypoderma*. Occasional abnormal infections of hosts other than bovids and cervids are observed (Minár 1987; Basiaga et al. 2014), including humans (Faber and Hendrik 2006; Puente et al. 2010; Panadero-Fontán and Otranto 2015).

The most common and most studied species are *Hypoderma bovis* and *H. lineatum* which mainly affect cattle (*Bos taurus*). *Hypoderma actaeon* is a typical parasite of red deer (*Cervus elaphus*) and strictly host-specific to it according to Brauer (1863). The main host of *Hypoderma diana* is the roe deer (*Capreolus capreolus*) but can also parasitize a wide range of hosts, *Cervus elaphus* (red deer), *Cervus nipon* (sika deer), *Dama dama* (fallow deer), *Alces alces* (elk), *Rangifer tarandus* (reindeer), *Rupicapra rupicapra* (chamois), *Ovis musimom* (muflon), (Zumpt 1965). *H. tarandi* has been recorded in reindeer (*Rangifer tarandus*) and finally *H. sinense*, subject of several scientific discussions, has been recognized as a valid species affecting cattle and yaks (*Bos grunniens*) in China (Otranto et al. 2004, 2005, 2006; Colwell 2001). In addition, Zumpt (1965) mentions two other species, *H. capreola* which has been considered as a synonym of *H. diana* and *H. moschiferi* of which only the first and second instar larvae are known and whose host-specific is the musk deer (*Moschus moschiferus*) but there is no other information.

*H. actaeon* is, so far, the only species found in *Cervus elaphus hispanicus* in Spain and, unlike other species, it seems that its life cycle takes place entirely under the skin without intra-organic migrations (Panadero et al. 2017; González et al. 2023). Its presence is very frequent and has been reported in different parts of North, Central and Southern of the country (Hernández et al. 1980; Gil-Collado et al. 1984; Martínez-Gómez et al. 1990; Pérez et al. 1995; Martínez-Moreno et al. 1997; De la Fuente-López et al. 2001; Domínguez et al. 2010; San Miguel et al. 2001; Panadero et al. 2017; González et al. 2023). Also, in Hungary (Sugar 1976; Husvéth and Egri 2021), Austria (Brauer 1863) and Portugal (Ahmed et al. 2017), with sporadic reports in cattle (Ahmed et al. 2017), fallow deer and roe deer (Panadero et al. 2010, 2017) despite its specificity.

Little is known about the pathogenic effects caused by *Hypoderma* species on wild cervids. It is assumed that, as in domestic animals, it affects their welfare and body condition, weakens the immune system, and increased susceptibility to diseases (Hassan et al. 2010; Ballesteros et al. 2012, Patra et al. 2018). In some cases, the infestation can be severe (Yeruham et al. 1994) and has been considered responsible for the death of wild deer during the winter (Fletcher 1987). Vicente et al. 2004 make some similar reflections with other Oestridae species.

After examining a large number of red deer with subcutaneous larvae on their backs, the aims of the present research were to describe the epidemiology of the parasite and to investigate the presence of anti-*Hypoderma* antibodies in serum samples by means of two different ELISA tests to better understand the chronobiology of the species in different climatic zones of the country.

## Materials and methods

### Study area

The study area was undertaken in the Riaño Regional Hunting Reserve (43° 03′ 14" N, 4° 57′ 33,85" W), Province of León, north-western Spain and located in the Cantabrian Mountains. The reserve occupies an area of 78,995 ha and the greater part of its territory is in the Atlantic region, where the landscape is considerably mountainous. The north is dominated by rocky alpine pastures while in southern predominate meadows and forests of *Quercus pyrenaica*, *Q. petraea* and *Fagus sylvatica* mainly.

The annual average temperature is 10.2ºC (-12ºC/36ºC); winter is long and cold and extends from October to April; December and January are the coldest months. Summer is short and cool with average temperatures of 16-17ºC. The mountains tops are frequently covered with snow and the difference in temperature between day and night may be as much as 20ºC. Rainfall is distributed throughout the year with an average annual precipitation of 1088.6 mm, reaching its maximum value in October and its minimum in July. Added to this is hidden precipitation, mainly in the form of cold dense fogs which are common in summer (AEMET, State Meteorological Station 2009).

Along with the wild animals of the area (red deer, roe deer, chamois (*Rupicapra rupicapra*), wild boar (*Sus scrofa*) and Spanish ibex), domestic animals coexist, mainly cattle and horse raised for meat, with sheep and goat farming in notable declive. In summer, the number of livestock in the area increases with the transhumance of cattle and sheep originating mainly of Extremadura region.

### Sampling procedures

Between March 2005 and March 2007, a total of 110 deer were studied, most of them were shot by the technical staff of the reserve in selective hunting. Ten animals in the winter of 2005 were found dead by Reserve personnel after heavy snowfall. For management reasons, it was not possible to obtain red deer during the summer months. The deer sampled were classified according to their sex, age groups (young ≤ 2 years old and adults > 2 years old), seasons [(W = winter (January, February, March); S = spring (April, May, June), A = autumn (October, November, December)], and years (2005 = 05, 2006 = 06, 2007 = 07), (Table 1).

**Table 1:** Number of animals according to their sex, age groups, seasons and years of sampling

Sampling	Host age		Sex		Nº deer
Season	≤ 2 years	> 2 years	Male	Female	examined
Winter 2005	2	13	8	7	15
Spring 2005	3	7	0	10	10
Autumn 2005	5	10	6	9	15
Winter 2006	2	13	1	14	15
Spring 2006	1	9	2	8	10
Autumn 2006	4	8	3	9	12
Winter 2007	3	12	4	11	15
Spring 2007	4	14	3	15	18
Total	24	86	27	83	110

After necropsy of the animals, the internal surface of the skin and the subcutaneous tissue were carefully examined. *Hypoderma* larvae, in all three stages, were found in the dorsal and lumbar regions of the animals, and their anatomical position was noted. The larvae were removed, counted, measured and preserved at -20ºC and in 70% ethanol for later study. Blood samples of 100 animals were taken by cardiac puncture or from the thoracic cavity. Sera was obtained by centrifugation, placed in vials, labelled and preserved at -20ºC until used for serological studies.

### Morphological identification of *Hypoderma* spp.

First, second and third-instar larvae were placed between two trichinoscopy plates to flatten them and then were mounted on a slide in temporary preparation with 0.05% lactophenol cotton blue stain or in semi-permanent preparations with glycerinated gelatin or in permanent preparations with a resin or synthetic resins. They were studied using a light microscope (Nikon YS-CF 100, Tokyo, Japan) and a stereomicroscope (Nikon SMZ 1500, Tokyo, Japan) according to Zumpt (1965) and Sugar (1976). The morphological identification of third-instar larvae was made according to Colwell et al. (1998) and Otranto et al. (2003a) of approximately 60% of the larvae collected, from different animals and years. For scanning electron microscopy observation (SEM, JEOL, JSM-6480-LV, Tokyo, Japan), third-instar larvae were separated into three portions using the posterior three segments and the anterior four segments for their study. Initially were classified by their size, shape, color and location under the fibrous connective tissue or inside subcutaneous nodules. Once the internal organs were removed, the samples were fixed, dehydrated with ethanol, dried with carbon dioxide and mounted on cylindrical metal supports, coated with a conductive silver glue and then metalized with gold. Their study took place mainly to obtain digital images and highlight their most relevant characteristics.

### Molecular analysis

The internal tissues of the mid-portion of five third-instar (V5-L3, V7-L3, V47-L3, V54-L3, V107-L3) and the entire larvae of two second-instar (V31-L2 and V34-L2.) and two first-instar (V82-L1, V90-L1) *Hypoderma* were used for DNA extraction digested overnight at 56ºC with 500 µl of lysis buffer mixed with 5 µl of proteinase K (20 mg/ml) (Merck). The day after, samples were centrifuged at 12.000 rpm for 5 min and the supernatants were collected. Genomic DNA was precipitated by adding isopropanol (Sigma Aldrich) and left dried at 56ºC for 1 h. The pellets were then resuspended in DNAse/RNAse-free distilled water (Thermofisher) and incubated at 37ºC for 30 min. DNA samples were quantified using a Nanodrop spectrophotometer (Thermofisher) and 50 ng of each sample were used for PCR amplification.

Cytochrome c oxidase subunit 1 (COI) gene was amplified using the pair of primers UEA7 (5΄-ACAGTTGGAATAGACGTTGATAC-3΄) and UEA10, (5΄-TCCAATGCACTAATCTGCCATATTA-3΄), previously described by Otranto et al. 2003b. Briefly, 1.5 U of proofreading *pfu* polymerase (Fermentas) was used to amplify 50 ng of genomic DNA in a final volume of 50 µl containing 1.5 mM MgCl_2_, 0,5 µM of each primer and 25 µM of each dNTPs. Samples were amplified in a TProfessional Basic Thermocycler (Biometra) using the following parameters: an initial denaturalization for 2 min at 95ºC, 40 cycles of 1 min at 95ºC, 1 min of annealing at 52ºC and 1 min at 72ºC, followed by a final extension of 7 min at 72ºC. The expected size of the PCR products was 688 base pairs. PCR products were subsequently analyzed in 1% low melting point agarose gels and bands were purified using the GeneJET Gel Extraction Kit (Thermofisher), following the manufacturer’s indications.

Purified PCR products were sequenced using Thermosequenase cycle sequencing kit in the sequencing DNA core facility of the University of León. Reference sequences obtained from Genbank corresponding to *Hypoderma bovis* (KT600293), *Hypoderma actaeon* (AF497765) and *Hypoderma lineatum* (KP965726) were compared and aligned with the nucleotide sequences obtained from our amplified samples using Clustal W (McWilliam et al. 2013). The sequences obtained from second and first-instars were compared only with *H. actaeon* (AF497765).

### Serological procedures

The presence of anti-*Hypoderma* antibodies (IgG) was investigated by an indirect enzyme-linked immunoassay (iELISA) using a crude larval extract (CLE) and a purified antigenic fraction, the hypodermin C (HyC), obtained from first-instar of Spanish isolates of *H. lineatum* (cattle) according to Panadero et al. 2010. The cut-off for the assay was calculated as the mean optical density (OD) of a population of negative sera from animals ≤ 2 years not infested with subcutaneous larvae plus two times the standard deviation (+ 2 SD). Positive absorbance values were established in OD > 0.237 for the CLE–ELISA and in OD > 0.263 for the HyC–ELISA.

### Statistical analysis

The prevalence and mean intensity of the infection were determined, considering host sex, age and sampling month, according to Bush et al. (1997). Data analysis was carried out using the chi-square non-parametric test in order to compare the prevalence, as well as the Mann–Whitney and Kruskal–Wallis tests to compare the intensity. The 95% confidence intervals (CI) for prevalence and mean intensity were calculated according to Martin et al. (1988). The median intensity and the aggregation index (variance/mean ratio) were calculated in order to observe the distribution of the subcutaneous larvae of *H. actaeon* (Rózsa et al. 2000). The Student´s t-test and ANOVA were used to determine differences in mean antibody levels. Correlations between OD values and the number of larvae were assessed using the Spearman´s rank correlation coefficient. The level of agreement among the three diagnostic methods (subcutaneous larvae, CLE-ELISA y HyC-ELISA) was assessed by the Kappa index (K). Statistical significance was determined at the P < 0.05 level. The statistical analysis was carried out using the SPSS 18.0 software package (SPSS Inc., Chicago, Illinois, USA).

## Results

### Morphological identification

**Fig. 1:**
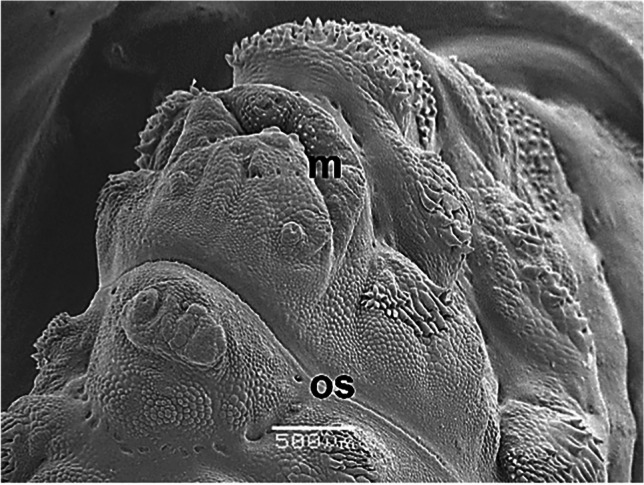
Third-instar *Hypoderma actaeon*. View of the cephalic segment. Note the absence of a band of spines between the mouth (m) and the opercular scar (os)

**Fig. 2:**
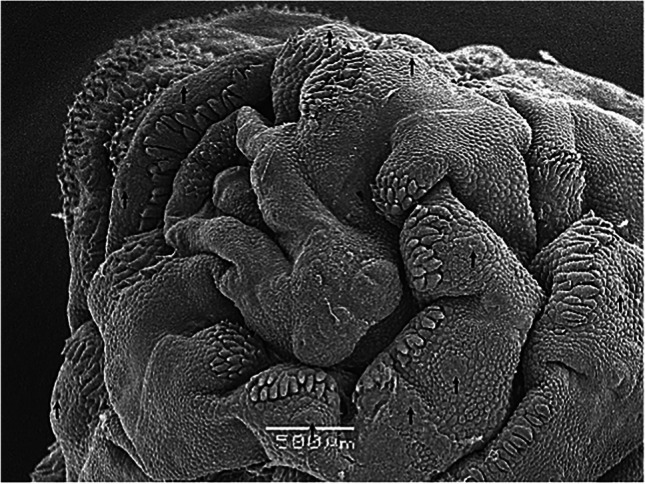
Cephalic segment showing the evagination of mounth hooks. Cuticular sensilla bellow each spine cluster are arrowed

**Fig. 3:**
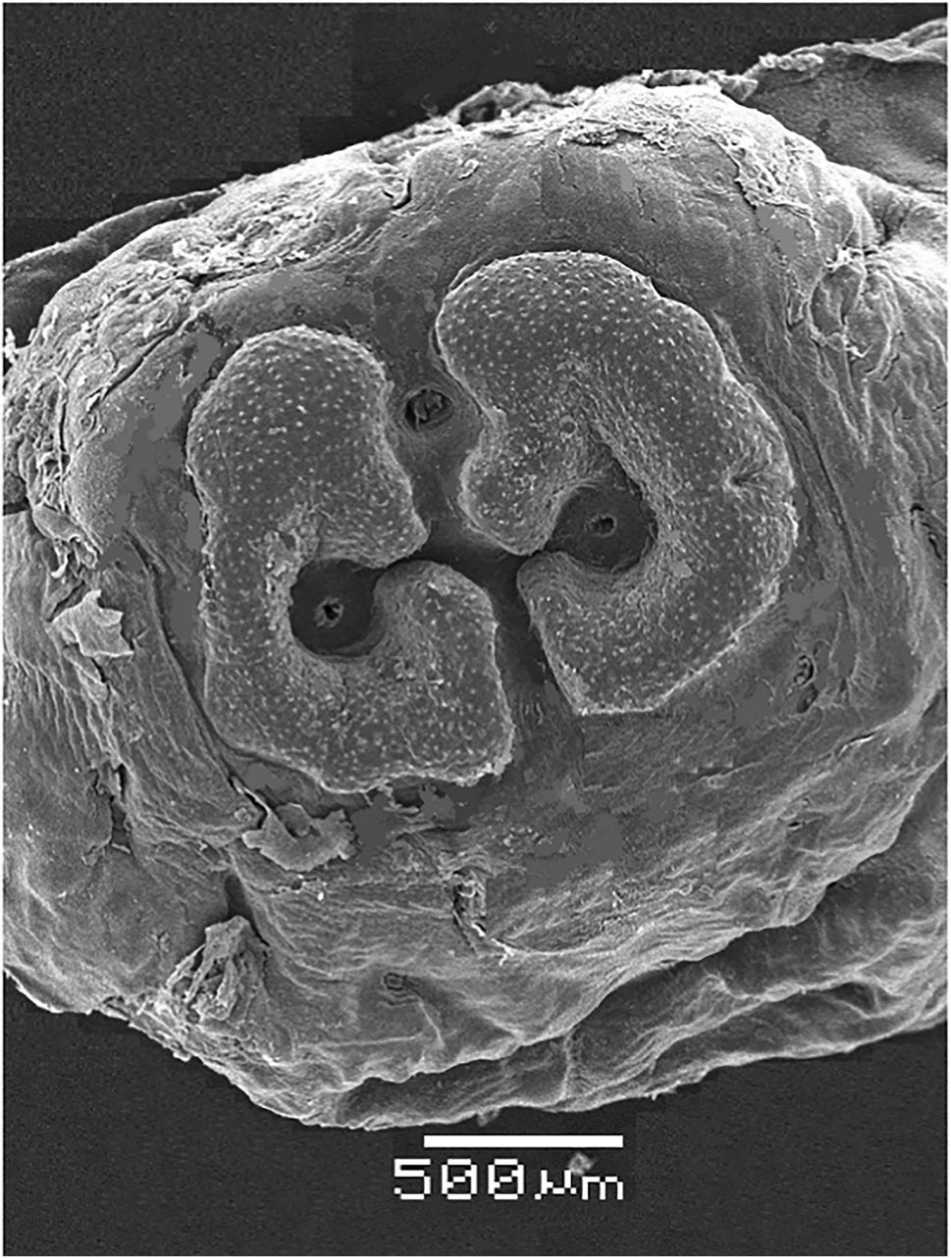
Spiracular plates in the last abdominal segment with the ecdysal scar in the center and surrounded by the spiracular plate (es: ecdysal scar; a: anus)

The external morphology of third-instar *H. actaeon* observed by SEM allows its differentiation from other species. Important distinguishing features include, in the cephalic segment, the absence of a band of spines between the oral opening and the opercular scar, the presence of a small band of spines ventral to the mouth and mouth hooks strong and well developed (Figs. 1 and 2). The thoracic segments have a double row of spines on their anterior and posterior margins. In the first, on the anterior margin, they are arranged in small groups and are associated with cuticular sensilla located below each one of them (Fig. 2). In the last abdominal segment, the spiracular plates are shaped like a "C" and its inverse, with the ecdysal scar in the center, completely surrounded by the plate and deeply sunken in it (Fig. 3).

The first- and second-instar larvae from this species, apart from their size, shape and location, do not present differential characteristics as clear as those of third-instar, although there are some peculiarities common to the three larval stages, such as the presence of a small band of spines ventral to the mouth, the lack of spination in the 10th abdominal segment and the existence of small denticles arranged randomly and covering the entire last abdominal segment.

### Molecular analyses

The direct sequencing of PCR products of five genomic DNA isolates from third-instars *Hypoderma* spp. yielded nucleotide sequences of 666 base pairs. When comparing them with the reference sequence of *H. actaeon* AF497765 deposited in GenBank, all of them exactly matched *H. actaeon* with a degree of interspecific difference of 0.291%. Subsequently the five isolates were compared with reference sequences from *H. bovis* (KT600293) and *H. lineatum* (KP965726), observing a degree of interspecific difference of 12.35% and 12.06%, respectively. The sequences were deposited in GenBank with accession numbers MZ696571, MZ700686, MZ700687, MZ700688 and MZ700689. Isolated samples from first- and second-instar larvae and blast analysis of mitocondrial COI sequences evidenced that the isolates corresponded with *H. actaeon* with a degree of interspecific difference of 0.3%. The sequences are also available in Genbank with the accession numbers ON540296, ON540297, ON540298 and ON540295.

### Epidemiological analyses

Subcutaneous larvae of *H. actaeon* were found in 47 of 110 red deer (42.7%, 95% CI: 33.4–51.9%) with a mean intensity of 12.5 ± 18 (range 1–80) warbles/infested host (95% CI: 7.3–17.6). Most hosts harbored less than 20 larvae (38/47; 80.9%) with a median intensity of 2.5 and a mean intensity of 5.13 ± 5.09 (95% CI: 3.5–6.8) larvae/host. The 59.3% of the total larval burden (392/587 larvae) was found in seven hosts (median intensity = 35, mean intensity = 43.55 ± 19.86, 95% CI: 30.6–56.5). Only one animal, a male older than 2 years, was infested by a large number of larvae (80 specimens). These data indicate that the distribution of larvae in the infested animals showed an aggregated/overdispersed pattern (aggregation index = 25.84), where the larvae are not randomly or uniformly distributed, but strongly aggregated among their hosts.

The adult animals had a higher prevalence (47.7%, 95% CI: 37.1–58.35%) than the young (25%, 95% CI: 7.67–42.3%) and the males (66.7%, 95% CI: 48.9–84.5%) higher than the females (34.9, 95% CI: 24.6–45.1%), finding statistically significant differences only according to the sex of the animals (χ^2^ = 8.38; *P* = 0.007).

Significant variations were observed when considering the seasons of the year (P = 0.002) with the highest number of infested animals in winter (62.22%, 95% CI: 48.1–76.4%), decreasing sharply in spring (26.32%, 95% CI: 12.3–40.3%) and a slight increase in the autumn (33.33%, 95% CI: 15.5–51.1%). No significant differences were observed between the sampling years.

When considering the mean intensity of the infestation, the previous pattern is repeated, with adult animals (13.4 ± 19, 95% CI: 9.4–17,4) and males (15.7 ± 21, 95% CI: 7.8–23.6) showing the highest values but without statistically significant differences. These differences were not found between the seasons of the year either, but they were observed between the sampling years (P = 0.04) (Table 2).

**Table 2:** Prevalence (%), mean intensity (± SD) of infection of red deer with subcutaneous larvae of *Hypoderma actaeon* and percentage of animals by iELISA using CLE and HyC as antigens* relative to host age, sex, seasons and year of sampling

Subctaneous larvae	Deer examined	Prevalence (%)	Mean intensity ± SD	Range	CLE (%)	HyC (%)
Host Age						
≤ 2 years	24	25	6.3 ± 6.2	1–14	65	35
> 2 years	86	47.7	13.4 ± 19	1–80	97.5	48.8
Sex						
Males	27	66.7	15.7 ± 21	1–80	92.3	50
Females	83	34.9	10.5 ± 15.9	1–61	90.5	44.6
Seasons						
Winter	45	62.2	15.9 ± 21.6	1–80	92.1	52.6
Spring	38	26.3	7.4 ± 10.5	1–35	85.7	31.4
Autumn	27	33.3	7.4 ± 7.2	1–21	96.3	55.6
Years						
2005	40	45	22.4 ± 24.5	1–80	90	55
2006	37	29.7	6.3 ± 7.4	1–23	94.1	52.9
2007	33	54.5	6.4 ± 8.7	1–35	88.5	23.1
Total	110	42.7	12.5 ± 18	1–80	91	46

^*^CLE and HyC calculated on 100 animals

A total of 587 larvae, in all three stages, were removed from the inner surface of the skin in the dorsal and lumbar regions of the animals. First-instars were found under a thin layer of fibrous connective tissue without observing any subcutaneous nodule or breathing hole to the outside. They are small, cylindrical slightly longer than wider and narrower ends, whitish or greyish, 5–10 mm long and 2–4 mm wide (7.4 ± 1.2 × 2.5 ± 0.7 mm; n = 20). They represented 15.6% (95% CI: 12.7–18.5%) of the total number of larvae found and were observed mainly in the autumn (29.1%; 95% CI:16.4–41.8%), progressively decreasing their presence during the winter (13.9%; 95% CI: 8.4–18.9%) and spring (9%; 95% CI: 1.4–16.6%).

Second-instar was found inside the warble observing the breathing hole. It is elongated and thin, rounded at the anterior end and the posterior sharper, 10–17 mm long and 3–5 mm wide (15.3 ± 1.4 × 3.5 ± 0.6 mm; *n* = 20) and yellowish to light brown in colour. They represented 35.3% (95% CI: 31.4–39.2%) of the total number of larvae found and showed the same pattern as first-instar, being found mainly in the autumn (70.8%; 95% CI: 57.9–83.7%) and their presence decreasing in winter (30.2%; 95% CI: 23.4–36.9%) and spring (21.6%; 95% CI: 10.8–32.4%).

**Fig. 4:**
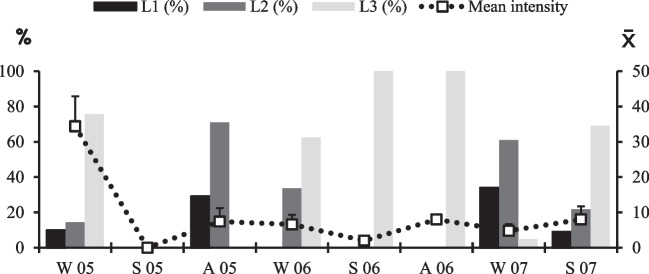
Chronobiology of the subcutaneous phase of *Hypoderma actaeon* in *Cervus elaphus* from Riaño Reserve, León, Spain (W = Winter; S = Spring, A = Autumn)

Third-instars were also found inside the warble with the peritreme in the breathing hole open in the skin. They are large and cylindrical, reaching 15–25 mm in length and 6–14 mm in width (20 ± 3 × 11 ± 2.3 mm) and ranged in colour from yellowish to completely black. They represented 49.1% (95% CI: 45.1–53.1%) of the total number of larvae found. Unlike first and second-instar, they were not observed in autumn, showing high values during winter (55.8%; 95% CI: 48.5–63%) and spring (69.4%; 95% CI: 57.2–81.5%) (Table 3 and Fig. 4).

**Table 3:** Percentage of larval stages of *H. actaeon* according to the seasons of the year

	L1 (%)	L2 (%)	L3 (%)
Winter	14.0	30.2	55.8
Spring	9.0	21.6	69.4
Autumn	29.1	70.8	-
Total	15.6	35.3	49.1

### Anti-Hypoderma antibody (IgG) pattern

The prevalence of seropositivity by iELISA was 91% for CLE, fluctuating between 80 and 100%, and 46% for HyC presenting a great variability of results throughout the study with statistically significant differences (13.3–80%, χ2 = 20.787; P = 0.008). Both tests detected antibodies in animals in which no subcutaneous larvae were observed (Table 4).

**Table 4:** Values of prevalence and mean intensity of subcutaneous larvae of *H. actaeon* with respect to seroprevalence and absorbance obtained by iELISA using CLE and HyC as antigens, according to the seasons and years of sampling

Season	Deer examined	LS (%)	CLE (%)	HyC (%)	LS	CLE	HyC
W-05	15	66.7	93.3	80	34.4 ± 26.9	2.339 ± 0.930	0.539 ± 0.281
S-05	10	-	80	20	0	2.033 ± 1.072	0.294 ± 0.272
A-05	15	53.3	93.3	53.3	7.4 ± 7.7	1.786 ± 1.030	0.286 ± 0.298
W-06	12	75	83.3	33.3	6.6 ± 8.2	1.523 ± 0.947	0.182 ± 0.158*
S-06	10	10	100	70	2	1.866 ± 0.670	0.633 ± 0.598
A-06	12	8.3	100	58.3	8	1.656 ± 0.910	0.322 ± 0.210
W-07	11	72.7	100	36.4	3.1 ± 3.0	1.559 ± 0.636	0.265 ± 0.303
S-07	15	46.7	80	13.3	7.0 ± 12.4	1.209 ± 0.671	0.219 ± 0.218*
	100		91	46			

^*^antibodies below the cut-off

The absorbance values ​​were higher with the CLE than with the HyC, and a significant positive correlation was found in the levels of antibodies between both serological tests (ρ = 0.598; P < 0.001). In general, these values gradually and with slight oscillations, decreased until the end of the study. The ANOVA test showed significant variations in the mean levels of antibodies throughout the study, using HyC as antigen (F = 2.777; P = 0.009), perhaps due to the fact that the mean level of antibodies was negative in two samplings, which was not observed when the CLE was used (Table 4).

Through Spearman's correlation, we observed that the number of larvae in infested animals and the mean level of antibodies was higher with HyC (ρ = 0.420; P = 0.005) than with CLE (ρ = 0.299; P = 0.049).

Similar to the finding of subcutaneous larvae, seroprevalence was higher in males and adults than in females and young animals, but only significant differences were found between the age groups with the CLE (χ2 = 20.635; P < 0.001) and also, the adult animals presented higher mean levels of antibodies than the young animals, also with the CLE (t = –5.186; P < 0.001). (Table 2).

**Fig. 5:**
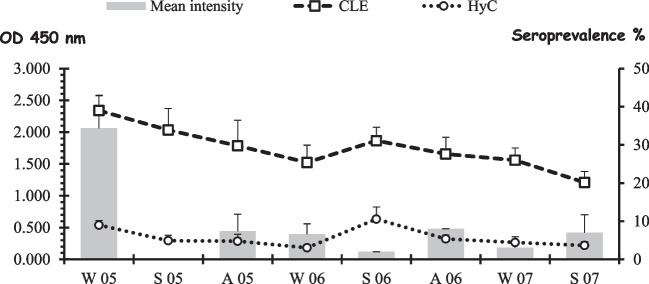
Seroprevalence (%) and mean intensity *anti-Hypoderma actaeon* in *Cervus elaphus* from Riaño Reserve, León, Spain (W = Winter; S = Spring, A = Autumn)

When comparing the three diagnostic methods used in the detection of *H. actaeon* infestation in red deer, we observed that of the animals that presented subcutaneous larvae, only one animal was negative by ELISA using both antigens and the rest of the animals were positive to serology, 23 for both antigens and 20 only for CLE. Only 8 animals were negative by the three methods, whereas 23 and 25, without subcutaneous larvae, were positive for both antigens or only for CLE, respectively (Table 5 and Fig. 5).

**Table 5:** Comparison of the results of the presence (L +) or absence (L-) of subcutaneous larvae with the iELISA using CLE and HyC as antigens

	L +	L-	Total
HyC + /CLE +	23	23	46
HyC + /CLE-	0	0	0
HyC-/CLE +	20	25	45
HyC-/CLE-	1	8	9
Total	44	56	100

The concordance between the presence of subcutaneous larvae and the levels of antibodies by means of the Kappa statistic was low (*κ* = 0.111 for HyC; *κ* = 0.108 for CLE) as well as between both ELISAs (*κ* = 0.155).

## Discussion

The morphological characteristics of the three larval stages found in this study and their molecular analysis confirm the identification of *H. actaeon*, the only species affecting red deer in northern Spain, as well as in the rest of the national territory. No evidence was found on the presence of *H. diana*, the other species of *Hypoderma* described in European deer (Sugar 1976). Despite its host specificity according to Brauer (1863), this species it is being diagnosed in roe deer from Spain in areas with high sympatry between roe and red deer (Panadero et al. 2017, 2020) and also has been found in cattle in Portugal (Ahmed et al. 2017), so the presence and transmission of other *Hypoderma* spp. to red deer is possible since this host normally coexists in the same area with other wild ruminants (*Capreolus capreolus*) and domestic livestock (*Bos taurus*). Thus, according to Price 1980, the species of flies would not present a strict host specificity and would rather depend on the availability of other hosts.

The life cycle of *H. actaeon*, without intraorganic migrations, makes it difficult to compare our results. Similarly, there is little information on the larval dynamics of *H. actaeon* in long-term studies. In Spain, only San Miguel et al. (2001) classified, in a very low number, some larvae as first-instar without giving further details. Our study revealed the presence of L1 and L2 mainly in autumn until the end of winter (November-March) and L3 in late winter until mid-spring (March–May), not finding infested animals in June, which means that all the larvae have already abandoned the host. This differs from what has been observed in the Mediterranean areas of the country and seems to indicate that in the north of Spain with colder temperatures during winter-spring–autumn, the consequence is the lower number of parasitized animals with a lower number of larvae per animal and total larvae, the emergence of the larvae from the back of the animals is delayed, the pupation period increases and the beginning of the activity period of the flies is extended to May–June, lengthening the life cycle approximately two months compared to the rest of Spain. Currently, the climatic changes that we are observing have a profound effect on parasite epidemiology of the parasite, with a direct influence on the development of the free-living stages of *Hypoderma*, hence our results coincide more with those observed by Sugar (1976) in Hungary and Brauer (1858) in Austria than in warmer areas.

As for other epidemiological aspects of the species, there are not many studies either and those that exist, the conditions in which they have been carried out vary considerably, so that their comparison is difficult and sometimes not very informative. In Spain, it has been studied in Mediterranean areas, in the center and south of the country, with very different approaches. In some points of these locations, the studies were limited to the hunting season of the animals, which spans from November to February, coinciding with the greater presence of L3 in the skin, so the prevalences obtained were much higher, as observed in the study by Pérez et al. (1995): 92% or Martínez-Gómez et al. (1990): 89%. In other areas where the studies covered the entire year, the prevalences found were considerably lower, 44.8% and 61%, according to De la Fuente-López et al. (2001) or San Miguel et al. (2001), respectively. However, Panadero et al. (2010) found a considerably low prevalence (14.2%) and concluded that the study would have been carried out when the larvae had already begun to leave the host.

In our study area, with an Atlantic climate, the prevalence found (42.7%) was similar to that found by De la Fuente-López et al. (2001), although we must bear in mind that these values could be different as we cannot sample the summer months due to technical problems. However, the differences were much more pronounced when considering the mean intensities. In our study, 12.5 ± 18, 1–80 larvae were found, values very different from those found by Pérez et al. 1995 (35.7 ± 41.3, 1–317) or De la Fuente-López et al. 2001 (38.29 ± 61.32).

When considering the age and sex of the animals, we found that adult animals (like Pérez et al. 1995 and Panadero et al. 2010) and males were more parasitized, with statistically significant differences between the sex of the animals. This differs from the findings in other hypodermosis studies where no differences between sexes were detected (Hurtado et al. 1997; Morrondo et al. 1999, Pérez et al. 1995) or where young animals (between < 1 year to 1–2 years of age) presented a much higher mean intensity of parasitism ($\overline{\mathrm{x} }$ = 100 larvae/animal), (De la Fuente-López et al. 2001; San Miguel et al. 2001).

In Europe, epidemiological studies of hypodermosis in red deer have only been carried out in Hungary, with prevalences much higher than those obtained in this study, ranging from 92.9% found by Sugar (1976), 79.4% by Husvéth and Egri (2021) and 67.9% by Kovács et al. (2011). In addition, this last author found that 100% of the males were parasitized compared to 70.1% of the females and the young animals were more than the adults.

The different parasitism between sexes is not very clear. Among the possible explanations would be in males the high levels of androgens could cause increased stress, which would cause an increase in the production of glucocorticosteroids and a decrease in acquired immunity and therefore an increase in the number of larvae parasitizing them (Folstad et al 1989). Other authors indicate different feeding habits, habitat use, pheromones or physiological variations between males and females. In the case of females, it would also be related to the increase in progesterone during pregnancy and prolactin during lactation of newborns. Both are immunosuppressive and are associated with increased susceptibility to parasitic infections. In the case of parasitism in adult and young animals, it would be related to their immune system.

The traditional diagnosis of livestock hypodermosis is based on palpation of the warbles on the back of the animals and in wild animals on its finding at necropsy, mainly during hunting seasons. In live animals serology is a useful tool. It is also useful in dead wild animals in epidemiological studies whenever blood samples are available. Thus, an ELISA was used for the first time by Engvall and Perlmann (1971) for the detection of antibodies in different diseases and has been the most widely used in the study of hypodermosis, especially in cattle. These methods are mainly focused on the detection of antibodies, one of them against the HyC, which is released by L1 of *Hypoderma* species, giving us more detailed information about the parasite-host interaction (Colwell et al. 2008). However, these tests present a series of problems such as the persistence of antibodies after the second year of infestation, after the larvae have emerged from the host or after they have died (Åsbakk et al. 2005; Panadero et al. 2006) so positive results only indicate that the animals have been exposed to the parasite but may not present an active infestation. Another problem is the lack of correlation between antibodies and the number of larvae present in the animals (Domínguez et al. 2010).

In our study, an ELISA test was used with CLE as antigen, observing a high prevalence (91%) and sensitivity (97.7%) but very low specificity (14.3%). The test detected antibodies in 43 of the 44 animals positive for the presence of subcutaneous larvae found at necropsy, and also in 48% of the animals where no grubs were observed. According to the findings of other studies in red deer and reindeer, this may indicate the persistence of antibodies in animals whose larvae have already exited the host (Panadero et al. 2010) or that the infestation is present but the larvae are not visible due to its small size and number, or because they die in the subcutaneous tissue, resulting in larval-negative animals (Åsbakk et al. 2014) or that they are false positives.

In principle, the high prevalence and sensitivity of the test seem to indicate that the flies are widely distributed in the north of Spain and with a well-established contact between the deer population and the flies. However, the low specificity found is not consistent with the high sensitivity and leads us to believe that the presence of antibodies may be produced by other parasitic processes present in the red deer at the same time as the hypodermosis and revealed in previous studies (Martínez et al. 2006; Diez-Baños et al. 2009; Hidalgo et al. 2010). Nevertheless, Sinclair and Wassall (1983), Monfray and Boulard (1990) and Boulard et al. (1996) among others, found no cross-reactivity of a certain number of parasites with *Hypoderma* spp. Perhaps, despite this, it would be desirable to check whether the presence of antibodies ​may be produced by cross reaction with other oestrids like *Pharyngomyia picta* and *Cephenemyia auribarbis* which coexist sympatrically with hypodermosis within this host (Bueno-de la Fuente et al. 1998).

Different results were found using HyC as antigen, observing a lower number of animals tested positively than with the crude antigen (46%) as well as the antibody mean values but more consistent with the prevalence found at necropsy (42.7%). Also showed a higher specificity than when using the previous antigen (58.9%) and a sensitivity (52.3%) more balanced. These results contrast with the findings of Panadero et al. (2010), where the prevalence was similar using both antigens (43.3% CLE vs 40.0% HyC), although their results with HyC are quite similar to those obtained by us.

In the case of the HyC ELISA, we observed that the mean level of antibodies was lower than with the CLE, even in two samples they were found antibodies below the cut-off in animals harboring subcutaneous larvae, which was also found by Panadero et al. (2010) in deer and Panadero et al. (2006) in cattle, stating that it may be due to the fact that the immunological system of these animals did not respond in a suitable way to the antigenic stimulus released by larvae. Also, the HyC ELISA was much more specific than the CLE, so the number of positively tested animals was much lower, but no concordance was observed between both methods or between the presence of subcutaneous larvae and antibody levels, which is manifested by the low values observed by means of the Kappa statistic. Since the antigen used comes from L1 of *H. lineatum*, perhaps it would be more desirable to obtain it from the L1 of the species that we want to analyze. Monfray and Boulard (1990) obtained the antigen from subcutaneous L1 and L2 of *H. tarandi* in reindeer with better results.

## Conclusions

The use of an indirect ELISA in the study of hypodermosis allows us to detect the infestation in live animals, to be able to establish an early treatment if necessary and allows us to know the epidemiology and biology of the species involved. In wild animals it allows to know in a short time the prevalence of the infestation without having to manipulate the skin or isolate one by one the larvae present, but it is not used for an early diagnosis or for the establishment of a treatment because this generally does not apply. Subsequently, with the morphological and molecular study, it will allow us to know the species involved in the hosts of the studied area, if the disease is spreading or remains stable or if the range of hosts is expanded, including mainly livestock and man. Thus, all these data suggest that hypodermosis is a prevalent myiasis in the Iberian Peninsula, although according to the National Wildlife Health Surveillance Plan (2022) it is not considered a relevant disease.

## Data Availability

The data that support the findings of this study are available in GenBank at https:// www.ncbi.nlm.nih.gov/genbank/, with the accession numbers: MZ696571, MZ700686, MZ700687, MZ700688, MZ700689, ON540295, ON540296, ON540297 and ON540298.
